# Purpura fulminans as the presenting manifestation of COVID-19

**DOI:** 10.1136/postgradmedj-2020-139202

**Published:** 2021-02-09

**Authors:** Ismat Ara Khan, Supratim Karmakar, Uddalak Chakraborty, Abheek Sil, Atanu Chandra

**Affiliations:** Dermatology, Medical College and Hospital Kolkata, Kolkata, West Bengal, India; Dermatology, Medical College and Hospital Kolkata, Kolkata, West Bengal, India; Neurology, Bangur Institute of Neurosciences, IPGMER & SSKM Annex 1, Kolkata, West Bengal, India; Dermatology, Venereology, and Leprosy, RG Kar Medical College and Hospital, Kolkata, West Bengal, India; Internal Medicine, RG Kar Medical College and Hospital, Kolkata, West Bengal, India

**Figure 1:**
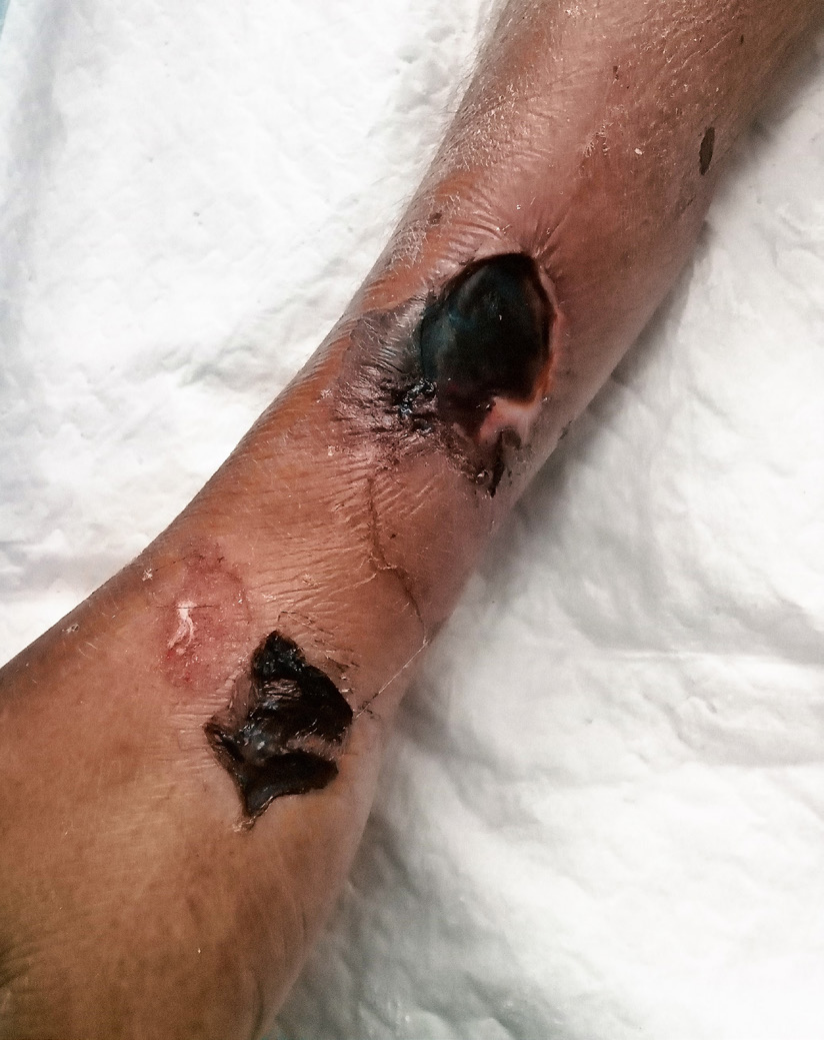
Multiple erythematous indurated plaques with focal necrosis, haemorrhagic bullae and peripheral retiform purpura.

A 48-year-old woman presented to our facility with a 2-day history of fever and rapidly enlarging skin discolouration over her right hand. On admission, normal vital parameters were noted. Cutaneous examination revealed two tender necrotic indurated plaques (5×4 cm and 10×6 cm) with focal haemorrhagic bulla and peripheral retiform purpura (figure 1). No abnormality was detected on systemic examination. Reverse transcriptase PCR for SARS-CoV-2 from oropharyngeal and nasopharyngeal swab was positive. Laboratory investigations documented thrombocytopenia (90×10^9^/L), elevated prothrombin time (17.6 s), prolonged activated partial thromboplastin time (53.5 s) along with elevated fibrin degradation products (37 mg/L; normal <10 mg/L) and D-dimer levels (7680 ng/mL; normal <500 ng/mL). Blood culture showed no growth. She was diagnosed with acute infectious (SARS-CoV-2) purpura fulminans and promptly started on anticoagulation therapy. Despite an initial improvement, the patient’s course of illness was complicated with severe acute respiratory distress syndrome and she died after 12 days of hospitalisation.

COVID-19 has been declared a pandemic by the WHO and has claimed innumerable lives until today. Though, majority of the cases present with respiratory symptoms, coagulation abnormalities and thrombosis in severe SARS-CoV-2 infection and its cutaneous manifestations are being increasingly recognised.^1^ Recently, livedoid and necrotic eruptions were noted in patients with more severe occlusive vascular disease.^2^ Purpura fulminans (PF) is a life-threatening disorder characterised by rapidly progressive cutaneous haemorrhage and necrosis caused by vascular thrombosis and disseminated intravascular coagulation. Three distinct categories identified include neonatal (inherited or acquired abnormalities of protein C, S or other coagulation systems), acute infectious (meningococcus, Gram-negative bacilli, *Staphylococcus* and varicella) and idiopathic.^3 4^ Physicians caring for patients with COVID-19 should recognise PF as a potential manifestation of underlying coagulopathy and immediately initiate anticoagulation therapy.
